# Persistent Hallucinations in a 46-Year-Old Woman After COVID-19 Infection: A Case Report

**DOI:** 10.7759/cureus.11993

**Published:** 2020-12-09

**Authors:** Tobechukwu A Clouden

**Affiliations:** 1 Psychiatry, Riverview Medical Center, Red Bank, USA

**Keywords:** delirium, hallucinations, covid 19, corona virus, psychosis, case study, case report, pandemic, visual hallucinations, persistent delirium

## Abstract

The coronavirus disease 2019 (COVID-19) pandemic has ravaged the world with its novel symptoms, infection rates, and death toll. The research community continues to learn more about the sequalae of the infection as it does not follow the pattern of a typical coronavirus. This is a case of persisting auditory and visual hallucinations in a 46-year-old woman after a COVID-19 induced delirium. The hallucinations remained, despite recovery from the virus, for almost three months with resolution of all other signs and symptoms. The hallucinations eventually disappeared and their persistence was believed to be continued symptomatology after treatment.

## Introduction

Coronaviruses are responsible for illnesses ranging from the common cold to severe upper respiratory infections. They also have neurotropic properties, and exposure may be associated with neuropsychiatric disease [1]. There have been increased corona virus immunoreactivity rates found in persons with new onset psychosis [1]. However, the coronavirus disease of 2019 (COVID-2019) does not follow the typical pattern of those in its class and has ravaged the world with the current pandemic. It has led millions to their deaths while many are plagued with severe physical complications. COVID-19 has been linked to other biological systems and the neurological sequalae includes brain inflammation, cognitive deficits, impairment of the senses, and hallucinations [2]. Should a delirium arise during its infection, atypical disturbances in attention, cognition and awareness may be observed.

Delirium is a direct physiological disturbance in an individual’s attention, cognition, and awareness [3-4]. Changes in cognition include memory deficits, language, and perception that fluctuate in severity throughout the day. It is a serious neuropsychiatric syndrome that is usually due to an acute medical condition, substance intoxication or withdrawal or toxin. Delirium affects up to 30% of hospitalized patients with an average duration of three to five days [5]. It is usually a transient phenomenon that subsides with treatment of the disease state.

Hallucinations are a common sensory misperception that can arise during the course of delirium. Visual hallucinations are the most common type of psychotic symptoms compared to delusions, auditory and tactile hallucinations [6]. Visual hallucinations in delirium have been associated with patients having increased medical disorders and deriving from multiple etiologies [6].

Persistent delirium, up to six months, can also occur [7]. Cognitive impairments and neurocognitive disorders are the strongest risk factors for delirium and the most common causes of persistent and severe delirium states [7]. Patients with neurodegenerative diseases, such as Alzheimer’s disease and Lewy body dementia, can have a delirium lasting over four months [7]. The elderly is at risk for persistent delirium compared to individuals of younger ages [7]. Individuals with cognitive impairments are usually elderly and such persons are the most vulnerable to severe and persistent delirium states.

Psychiatrists are being consulted to manage persistent delirium in COVID-19 patients. These patients demonstrate common risk factors such as advanced age (typically 75 years old), male and with cognitive decline [8]. There are proposed reasons for this condition including lack of touch, lack of visitors, no faces to read due to masks, and staff fatigue [8]. This case report presents a 46-year-old woman with no pre-existing medical conditions who developed a persisting delirium after treatment for COVID-19.

## Case presentation

This is a 46-year-old Spanish speaking woman with no known medical conditions who went to the ER with several complaints including fever, chest pain, vomiting, and cough. A family member also noted that she was becoming more forgetful and confused recently. She reported having the symptoms for approximately two weeks before going to the hospital. She was suspected of contracting COVID-19 and was tested. Chest X-ray demonstrated possible early pneumonia. She was diagnosed with pneumonia, most likely secondary to COVID-19 and was sent home with hydroxychloroquine and azithromycin. The following day she was called, informed of a positive test, and told to self-quarantine in her home for 14 days.

She returned to the ER eight days later with the same complaints in addition to abdominal pain, vomiting, and diarrhea. She developed shaking chills while in the ER and started having visual hallucinations of man telling her to come with him. Her lab tests demonstrated mild leukocytosis, hypokalemia, and elevated lactic acid levels. Her chest X-ray and CT scan of abdomen and pelvis did not demonstrate any abnormal findings. MRI of the brain was negative for any acute pathology including masses or vascular abnormalities. There was no known source of infection. She was started on vancomycin and imipenem and admitted for supportive care and hydration. Psychiatry was consulted for her hallucinations; she was diagnosed with delirium most likely secondary to COVID-19 and prescribed risperidone 1.5 mg twice a day. When stable, she was discharged home with psychiatry and internal medicine follow up appointments. She was instructed to remain in quarantine when home.

She met with the psychiatrist nine days later via a telehealth appointment. The outpatient psychiatric facility was not seeing patients in person due to COVID-19. She reported physical improvement but the hallucinations from the hospital remained. She saw a man in scrubs who stood in the corner of the room. His face was blurry but she suspected he was Caucasian. He was constantly saying, “I am here for you," in English. His presence was constant, and he never disappeared. No one else in the house could see him. She remained quarantined in her room all day, only leaving to use the bathroom. He did not follow her to the bathroom but was always standing in the corner when she returned. She was unable to sleep for more than an hour fearing he would harm her. She also reported having extreme financial difficulties due to unemployment. Her employer had to close due to COVID-19. She was also in the middle of a divorce and her husband was incarcerated. She had started seeing a therapist due to her social stressors. She agreed to symptoms of anxiety and depression including feelings of worthlessness, impaired concentration, constant worry, and fleeting passive suicidal ideations. She denied panic attacks, mania, vision impairments, other psychotic symptoms, and substance use.

Her mental status exam was limited given sessions were done via telephone. She had a strong Spanish accent. Her voice was calm and pleasant, and she was extremely cooperative during the interview. Her speech was productive with normal volume. Her mood was “okay,” and thought process was linear. She reported visual and auditory hallucinations. Her memory was grossly intact, and insight and judgement were appropriate. The psychiatrist added a diagnosis of major depressive disorder, mild with anxious distress. The hallucinations were attributed to delirium. The psychiatrist prescribed trazodone 50 mg nightly and haloperidol 2 mg nightly. Haloperidol was given in place of risperidone as it was too costly for her.

The psychiatrist spoke to her outpatient therapist. She reported their sessions began approximately five months prior to her hospitalization. She explained the patient’s traumatic history including sexual, physical, and emotional abuse by her husband and a diagnosis of post-traumatic stress disorder (PTSD). The therapist denied symptoms of depression, psychosis, or any severe mental decompensation during their sessions. She was of stable mental status, usually well groomed, calm and with good eye contact. She described her to be a pleasant woman who was trying her best to deal with her ongoing stressors and history of trauma. She was an active participant in the therapy with good insight. She only started reporting hallucinations after discharge from the hospital. The patient provided the same description of the hallucinations to the therapist. Otherwise her mental status had not changed. The therapist inquired further about the signs and symptoms of psychotic disorders. With the psychiatrist’s education, she, again, completely denied such findings in the patient. 

The hallucinations were still present at her following three-week appointment. The hallucinations persisted with no difference in intensity or frequency. She had only achieved an additional hour of sleep since the last visit and trazodone was increased to 100 mg.

The following week she was complaining of bilateral lower extremity edema, swelling of her hand, and coughing up mucus. The man was still talking to her and saying, “I’m coming to get you.” She had remained quarantined as instructed. She had not seen an internist since discharge from the hospital and was put on the waiting list at the family health clinic. The psychiatrist instructed her to go to the hospital and she was admitted the same day. Her COVID-19 test was still positive, 39 days since her last test. The remaining labs were all normal and a second MRI of the brain found no abnormalities. She was seen by the consulting psychiatrist and discharged on risperidone twice a day, haldol 1 mg nightly, and trazodone 200 mg nightly. She was discharged two days later and instructed to continue self-quarantine.

Nine days later she met with the outpatient psychiatrist. The hallucinations remained with no change in intensity, frequency, or description. She continued to have sleep impairment due to fear of the man and constant worries about her social stressors. She tried to avoid paying attention to him, but he was always there. The psychiatrist called the family health outpatient clinic given the urgency of follow up and was given an appointment. Two weeks later she reported some improvement in sleep as she was learning to accept the man’s ongoing presence. She remained depressed and anxious with the continued quarantine, unemployment, hallucinations, and financial constraints. She now had an additional stressor of finding a new place to live. She and her family could not pay the rent and had to leave their residence. The psychiatrist changed the risperidone to quetiapine 25 mg nightly and paroxetine 20 mg daily to aid with the sleep, hallucinations, depression, and anxiety.

The psychiatrist was able to speak with the outpatient internal medicine physician following the patient’s appointment. He also noted the patient to be quite lucid without any objective signs of mental deterioration or psychosis. He found the case to be extremely bizarre, but he and the psychiatrist agreed the hallucinations stemmed from the COVID-19 infection.

She reported being COVID-19 negative at the next session. This was approximately two months after her initial positive test. She was given the test at the family health outpatient facility. She could only verbalize partial relief as the man was still present. The psychiatrist asked for more details related to the hallucinations. She explained how the man follows her everywhere. He remained by her side the second time she went to the hospital. He was in the car, in the ER, next to her in the hallways, in the MRI suite and hospital room. Once she returned home, he entered the room and returned to the corner. He does not follow her to the bathroom but is always in the corner of the room upon her return. He does not yell, belittle her, or comment on her daily activities. He now spoke, occasionally, in English, asking her to go with him. Her sleep improved to approximately six hours at night, but she requested a higher dose of quetiapine for hallucinations. She continued to worry about her social stressors, but was more hopeful and optimistic. She had found another place to live and wondered if the man would follow her to the new residence. The quetiapine was increased to 50 mg and paroxetine remained at the same dose.

The psychiatrist spoke with the patient 13 days later to learn the hallucinations had completely disappeared. She had not seen the man for a week and was quite excited and relieved. She was sleeping well and remained optimistic. She was also excited about her new residence. It was in an urban setting with access to more employment.

## Discussion

This case report demonstrates how delirium can emerge from an acute illness, in this case, COVID-19 infection. The patient’s delirium began in the ER when she started having chills and hallucinations. The course of the delirium symptoms followed the typical trajectory, however, the hallucinations persisted approximately 15 weeks after treatment. This offers a unique distinction from the usual case of delirium as the patient was a female, did not have any physical comorbid illnesses, was not cognitively impaired or of advanced age. 

There was little possibility of re-exposure to the illness or another delirium state. She remained in self-quarantine upon hospital discharge. When she was asked to go to the hospital the second time, there was no indication that she had acquired another infection. Her chest X-ray and labs were all normal. Her reported edema and mucous-filled expectorant were most likely due to the initial COVID-19 infection. She completed a full work up with a second MRI that did not exhibit any significant findings.

The patient did neither have a history of a psychotic illness nor were the hallucinations thought to be a newly acquired psychotic disorder. She was diagnosed with PTSD only a few months prior to the infection while a mild major depressive disorder was identified after discharge. Corona viruses have been associated with new onset psychosis, however, the patient remained with a lucid sensorium and intact reality testing. The course of hallucinations primarily due to psychiatric disease does not allow for such stable mentation. Also, her chief complaint was a visual hallucination, which is the most common psychotic symptom associated with delirium. Such hallucinations are associated with a greater number of acute illnesses. However, the patient had no other ailments aside from the COVID-19 infection. 

The delirium itself was unique in that the hallucinations were the only persisting symptoms. Individuals with persisting delirium usually have continued fluctuating cognition, severe aggression, restlessness, disinhibition, and hallucinations. The patient remained with the one predominant symptom of visual hallucinations. 

COVID-19 played a significant role in the development of the patient’s delirium but the impact of psychiatric disease and psychological stressors cannot be ignored. She was free of physical comorbid illnesses but was suffering from the psychological disorders of PTSD and major depressive disorder. These disorders are linked to neurochemical alterations in the body that could play a role in the vulnerability to physical disease states. Her ongoing psychological stressors could have increased her risk to delirium due to associated biochemical imbalances. 

## Conclusions

Prior to this case, persistent delirium has been diagnosed in the presence of advanced age and/or neurocognitive decline. There are no known studies that have reviewed such a delirium without these risk factors. We learn from this case that persistent delirium can occur in an individual with no physical co-morbidities. However, the caveat to this report is the patient’s infection with COVID-19. The study supports the idea that psychological illness can present a vulnerability to acute disease states similar to physical co-morbidities. A COVID-19 infection may be the only risk factor needed for delerium and persistent delirium in patients with psychiatric disease. Further studies could explore the emergence of COVID-19 induced delirium in psychiatric patients who are otherwise healthy. Studies could also examine the symptomatology and persistence of symptoms in such individuals. 
